# Occurrence and Risk Assessment of Fumonisin B_1_ and B_2_ Mycotoxins in Maize-Based Food Products in Hungary

**DOI:** 10.3390/toxins11120709

**Published:** 2019-12-05

**Authors:** Andrea Zentai, Mária Szeitzné-Szabó, Gábor Mihucz, Nóra Szeli, András Szabó, Melinda Kovács

**Affiliations:** 1 System Management and Supervision Directorate, National Food Chain Safety Office, 1024 Budapest, Hungary; zentaia@nebih.gov.hu; 2 Faculty of Agricultural and Environmental Sciences, University of Kaposvár, Guba S. u. 40., 7400 Kaposvár, Hungary; dr.szabomaria@gmail.com (M.S.-S.); mihucz.gabor@ke.hu (G.M.); szeli.nora@ke.hu (N.S.); kovacs.melinda@ke.hu (M.K.); 3 MTA-KE-SZIE Mycotoxins in the Food Chain Research Group, Kaposvár University, Guba S. u. 40., 7400 Kaposvár, Hungary

**Keywords:** fumonisin, human exposure, maize products

## Abstract

Fumonisins are toxic secondary metabolites produced mainly by *Fusarium verticillioides* and *Fusarium proliferatum.* Their toxicity was evaluated, and health-based guidance values established on the basis of both Joint FAO/WHO Expert Committee on Food Additives (JECFA) and European Food Safety Authority (EFSA) recommendations. This study presents the results of fumonisin analyses in different maize- and rice-based food products in Hungary and the potential health risk arising from their dietary intake. In total, 326 samples were measured in 2017 and 2018 to determine fumonisins B_1_ and B_2_ levels. Three-day dietary record data were collected from 4992 consumers, in 2009. For each food category, the average concentration values were multiplied by the relevant individual consumption data, and the results were compared to the reference values. With respect to the maximum limits, one maize flour, two maize grits, and two samples of other maize-based, snack-like products had total fumonisin content minimally exceeding the EU regulatory limit. The mean daily intake for all maize-product consumers was 0.045–0.120 µg/kg bw/day. The high intake (95 percentile) ranged between 0.182 and 0.396 µg/kg bw/day, well below the 1 µg/kg bw/day tolerable daily intake (TDI) established by EFSA. While the intake calculations resulted in comforting results, maize-based products may indeed be contaminated by fumonisins. Therefore, frequent monitoring of fumonisins’ levels and evaluation of their intakes using the best available data are recommended.

## 1. Introduction

Fumonisins are secondary metabolites produced mainly by *Fusarium verticillioides* and *Fusarium proliferatum* [1]. Maize and maize-based products are most commonly contaminated by fumonisins, but fumonisins can be detected in several other cereal grains, such as rice, wheat, barley, rye, and oat [2,3]. More than 15 fumonisin homologues have been described, including fumonisin A, B, C, and P, and, among them, fumonisin B_1_ (FB_1_), FB_2_, and FB_3_ are the most frequent naturally occurring fumonisins [1,4]. FB_1_ typically accounts for 70%–80% of the total fumonisin produced, while FB_2_ usually makes up 15%–25% and FB_3_ 3%–8% when cultured on maize, rice, or in liquid medium [1].

Among fumonisins, FB_1_ is the most toxic compound and has been shown to promote tumour growth in rats as well as equine leukoencephalomalacia [5] and porcine pulmonary oedema [6]. It was classified by the International Agency for Research on Cancer (IARC) in Group 2B (possibly carcinogenic in humans) [7]. FB_1_ also causes chronic liver and kidney toxicity when administered in repeated doses to rodents.

Fumonisin B toxins, as structural analogues of sphingoid bases, inhibit ceramide synthases, causing the disruption of the sphingolipid metabolism and leading to sphinganine (and sphingosine) accumulation in cells and tissues [8]. Toxicity studies have mainly focused on the effects of FB_1_, but FB_2–4_ appear to have similar toxicological profiles. Acute toxicity is not relevant for fumonisins.

The Scientific Committee on Food (SCF) as well as the European Food Safety Authority (EFSA) in Europe and the Joint FAO/WHO Expert Committee on Food Additives (JECFA) evaluated the dietary risk of fumonisin intakes [9,10,11,12,13,14,15].

The SCF established a tolerable daily intake (TDI) of 2 µg/kg bw/day for FB_1_ in 2000, based on an overall level of no observed adverse effect (NOAEL) of 0.2 mg/kg bw for liver and kidney in rodents [9]. This TDI was expanded into group TDI in relation to the total amounts of fumonisin B_1_, B_2_, and B_3_, alone or in combination [10]. JECFA published a risk assessment on FB_1_, FB_2_, FB_3_ in 2001 [11]. The assessment was essentially based on FB_1_ data, and the other toxins were considered as having similar toxicological profiles. A group provisional maximum tolerable daily intake (PMTDI) of 2 µg/kg bw/day per day was allocated based on a NOAEL of 0.2 mg FB_1_/kg bw per day for renal toxicity in a subchronic and a chronic rat study [11]. The PMTDI established by JECFA was retained in 2011 and in 2016 as well [12,13].

EFSA discussed food safety issues of mycotoxins, including fumonisins, in several documents. The chemical structure of mycotoxins can be altered by the defense reaction of plants, rendering them extractable conjugated and/or non-extractable bound mycotoxins or mycotoxin metabolites. Since these modified toxins are usually not detected during the analysis of mycotoxins, they are commonly termed “masked” or “bound”. EFSA issued a scientific opinion in 2014 regarding certain modified mycotoxins in food and feed [14].

More recently, EFSA published a scientific opinion on the appropriateness to set a group health-based guidance value for fumonisins and their modified forms in 2018 [15]. For the establishment of the TDI, the benchmark dose lower confidence limit (BMDL10) of 0.1 mg/kg bw per day for induction of megalocytic hepatocytes in mice was used. Taking into account an uncertainty factor (UF) of 100 for intra- and interspecies variability, the TDI was established at 1.0 µg FB_1_/kg bw per day. FB_2_, FB_3_, and FB_4_ were included in the TDI, based on structural similarity and the limited available data indicating similar mode of action (MoA) and toxic potencies.

In Europe, Commission Regulation (EC) No 1881/2006, setting the maximum levels for certain contaminants in foodstuffs, established the maximum limits for fumonisins (sum of B_1_ and B_2_) in different commodities, including unprocessed maize, maize intended for direct human consumption, maize-based foods for direct human consumption, maize-based breakfast cereals, maize-based snacks, processed maize-based foods and baby foods for infants and young children, different milling fractions of maize, and other maize milling products not used for direct human consumption. Table 1 presents the specified maximum limits by commodities.

This article presents the results of fumonisin analyses in different maize- and rice-based food products in Hungary and, consequently, the potential health risk arising from their dietary intake.

## 2. Results and Discussion

### 2.1. An Overview of the Measured Fumonisin Content

Altogether, 326 samples were measured for fumonisins B_1_ and B_2_ mycotoxins levels. The types of samples were from the food categories of maize flour, maize grits, corn flakes, canned maize, other maize-based, snack-like products, white and brown rice, and other rice-based products. The limit of detection (LOD) and the limit of quantification (LOQ) for FB_1_ were 0.031 and 0.093 mg/kg, while those for FB_2_ were 0.051 and 0.154 mg/kg.

In total, 70 and 256 samples were analyzed in 2017 and 2018, respectively, and were considered together in our assessment.

We measured 64 maize flour samples, of which 33 (51.6%) had detectable FB_1_ content, and 6 (9.4%) had detectable FB_2_ content. The highest FB_1_ value was 1.46 mg/kg. The average FB_1_ and FB_2_ concentrations were 0.17–0.20 mg/kg for FB_1_ and 0.05–0.10 mg/kg for FB_2_. In no instance was FB_2_ detected if FB_1_ was undetected. Only in six cases, both FB_1_ and FB_2_ were detected at a measurable level (above LOQ), while FB_2_ was never detected alone.

Then, 62 maize grits were analyzed; 26 samples (41.9%) presented detectable FB_1_, and 4 (6.5%) detectable FB_2_. The highest concentrations found were 1.96 mg/kg for FB_1_ and 0.58 mg/kg for FB_2_. The average FB_1_ content was 0.13–0.16 mg/kg, while the average FB_2_ content was 0.03–0.08 mg/kg. Four samples contained both FB_1_ and FB_2_ above the LOQ.

Altogether, 8 of the 64 corn flakes samples (12.5%) had measurable FB_1_ content, whereas FB_2_ was not detectable in any of them. The average fumonisin B_1_ content ranged between 0.03 and 0.07 mg/kg, and the highest measured value was 0.46 mg/kg.

Only one of the 18 canned maize samples contained measurable FB_1_, but none of them contained FB_2_. The relevant FB_1_ concentration was 0.20 mg/kg.

Fumonisin B_1_ was measured in 20% of the other maize-based, snack-like products (17 of the 85 samples), and FB_2_ in only 2 samples. The average FB_1_ content ranged between 0.07 and 0.10 mg/kg, with a maximum content of 1.1 mg/kg.

Regarding white rice and brown rice samples and other rice-based products, FB_1_ and FB_2_ contents were in all cases below the LOQ. These commodities were therefore not included in our further risk assessment. The most important parameters of the analysis results for each food categories are summarized in Table 2 and Table 3.

Considering these results in light of the current maximum limits, one maize flour, two maize grits, and two samples of the other maize-based, snack-like products (mexicorn and a maize wafer) had total fumonisin contents minimally exceeding the regulatory limit (the sum was calculated according to the upper-bound (UB) scenario in case of a non-detectable value of FB_2_).

Our results were also compared with fumonisin contents measured and published in the previous decades in Hungary. Fazekas et al. [16] measured considerably high fumonisin concentrations in maize collected during storage and harvesting in 1993 and 1994. Of the moldy maize samples collected in the period of storage, 70.8% contained fumonisin B_1_ (0.05–19.8 mg/kg; average concentration: 2.6 mg/kg). Fumonisin B_1_ content measured in maize ears more or less affected by molds (affected sample), collected in the period of harvesting, ranged between 0.095 and 52.4 mg/kg, with an average content of 6.64 mg/kg in 70% of the samples. Of the “average samples”, 30% were contaminated with fumonisin B_1_ (0.06–5.1 mg/kg; average: 1.52 mg/kg). Fumonisin concentrations were determined by high-performance liquid chromatography methods.

Tóth et al. [17] investigated *Aspergillus* and *Penicillium* species and their mycotoxins in maize in Hungary in two consecutive years after harvest. Mycotoxin concentrations were measured with HPLC–MS technique. Fumonisins (B_1_ + B_2_) were observed in quantities exceeding the EU limit in some samples collected in different regions (4.66 mg/kg; 10.15 mg/kg; 5.13 mg/kg; 7.55 mg/kg) in 2010.

The IARC report cites contamination data in maize for Europe, including Hungary. Fumonisin B_1_ was detected in 248 out of 714 maize samples, at a concentration range of 0.007–250 mg/kg [7]. Similarly, the WHO series of Environmental Health Criteria dealt with fumonisin B_1_ in 2000 [18]. The report specifically cites the results of the Hungarian authors Fazekas et al. [19], measuring 0.05–75.10 mg/kg fumonisin B_1_ in 56 out of 92 maize samples.

Comparing our results with those of the above reports, fumonisin contamination in Hungary in recent years seems to be lower than that measured in previous decades. However, our measurements focused on processed food products (targeting the end consumer), which obviously have lower fumonisin contents than unprocessed maize samples.

### 2.2. Correlation between FB_1_ and FB_2_ Levels

FB_2_ content was always lower than FB_1_ content in our samples and was detected only in those samples also containing FB_1_. The relationship between fumonisin B_1_ and B_2_ contents was further analyzed, to understand whether a possible correlation coefficient could be set up.

The commodity groups of at least one sample containing measurable quantities of FB_1_ and FB_2_ together were maize flour (six samples), maize grits (four samples), and the other maize-based snacks (two samples). The correlation coefficient calculated for the maize flour commodity group based on the numerical concentrations was 0.95, indicating a strong correlation.

Taking into account all 35 samples where, beside FB_1_, FB_2_ was also detected but not measurable (i.e., between LOD and LOQ), the correlation coefficients were 0.79 and 0.77 in the lower-bound (LB) and UB scenarios, respectively. Considering only the pooled maize flour and maize grits samples (26 samples), the correlation coefficient values were 0.86 and 0.82 in the LB and UB scenarios, respectively.

These results suggest a possible correlation between the levels of fumonisins B_1_ and B_2_; however, a higher number of samples with measured fumonisin B_1_ and B_2_ concentrations would be necessary to draw further conclusions.

### 2.3. Risk Assessment

The resulting intake values—both mean and high percentile—were well below the reference values established by EFSA and JECFA. Table 4 presents the calculated population mean and 95 percentile intakes for the five commodity groups (maize flour, maize grits, corn flakes, canned maize, and other maize-based, snack-like products) concerned.

The mean daily intake for all maize-product consumers based on the LB and UB scenarios was 0.045–0.120 µg/kg bw/day. In addition, the high intake (95 percentile) ranged between 0.182 and 0.396 µg/kg bw/day, well below 1 µg/kg bw/day.

Regarding children (aged 0–18 years), the mean intake was 0.056–0.167 µg/kg bw/day, and the high intake (95 percentile) was 0.244–0.537 µg/kg bw/day.

**Figure 1:**
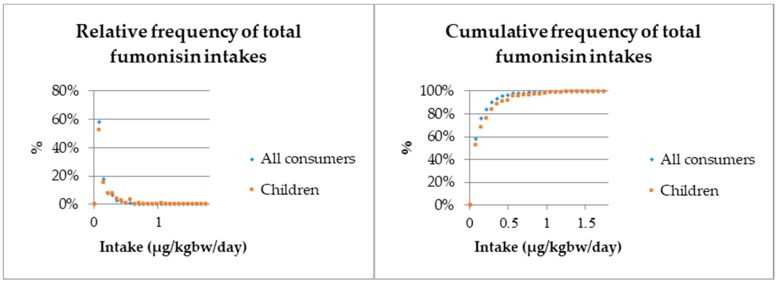
Relative and cumulative frequencies of total fumonisin intakes derived from maize-based products.

Figure 1 presents the relative and cumulative frequencies of the resulting distributions of total fumonisin intakes for both total consumer population and children. The figure shows that most intakes cumulated below 0.5 µg/kg bw/day.

The results were compared to those of the exposure assessment conducted by EFSA in 2014 on the occasion of a derogation request for the maximum levels of several mycotoxins, including fumonisins [20]. On the basis of French contamination data of 2013, the mean exposure levels in children groups ranged between 0.17 and 1.52 µg/kg bw/day in the LB scenario and between 0.47 and 2.11 µg/kg bw/day in the UB scenario. The high (95 percentile) exposure levels ranged between 0.54 and 3.44 µg/kg bw/day and between 1.09 and 4.39 µg/kg bw/day in the LB and UB scenarios, respectively. In adult groups, the mean exposure levels were between 0.03 and 0.81 µg/kg bw/day in the LB scenario and between 0.15 and 1.19 µg/kg bw/day in the UB scenario. The 95th percentile, however, ranged between 0.08 and 1.76 µg/kg bw/day in the LB scenario and between 0.31 and 2.30 µg/kg bw/day in the UB scenario.

Our present results are in the same range or—especially in the case of children—considerably lower than reported results (Table 5).

Although the estimated mean and high intakes remained below both the JECFA and the EFSA reference values in all scenarios, it is worth noting that the maximum and some high values (over the 95 percentile) exceeded the 1 µg/kg bw TDI set by EFSA in 2018. In the case of all consumers, these high values amounted to 0.97% of the population, whereas in the case of children, they amounted to 2.36%. The maximum estimated intake value was 1.81 µg/kg bw. These specific high values were predominantly children’s intake values, derived mainly from the consumption of canned and sweet maize and other maize-based snack-like products.

Considering that these intake results are based on the actually registered consumptions, representing only 4.8% of the total population and 7.6% of children consumers, the consequent health risk is probably negligible.

### 2.4. Commodity Contributions

The contributions of different commodities to the summed intake estimated from all maize-based foods are presented in Table 6.

**Figure 2:**
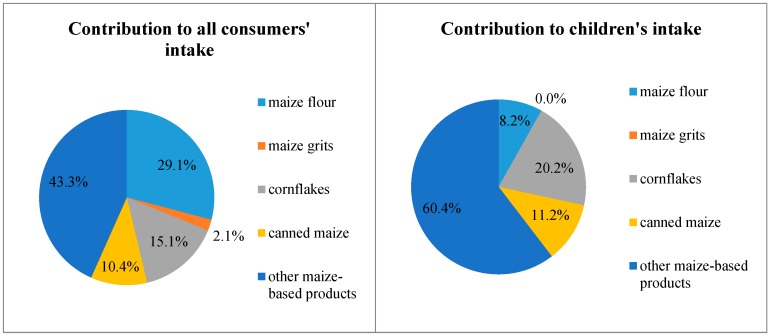
Contribution of food commodities to total fumonisin intake from maize-based products.

Considering the LB scenarios, maize-based, snack-like products contributed the most to the fumonisin intake of the total (all consumers) population (43.3%), followed by maize flour (29.1%) and corn flakes (15.1%). In the case of children, the main contributors in the LB scenario were, similarly, maize-based, snack-like products (60.4%), corn flakes (20.2%), and canned maize (11.2%) (see Figure 2).

### 2.5. Uncertainty Considerations

It should be mentioned that this assessment focused only on the intake of fumonisins B_1_ and B_2_ from five different maize-based commodity types. Other types and the modified or masked forms of fumonisins were not analyzed. Total fumonisin intake of the population could be somewhat higher, if all relevant (including also non-maize-based) commodity types were considered. However, given that maize is the focal commodity in relation to fumonisin contamination, the contribution of other food products to total fumonisin intake is considered low.

The effect of household food processing on fumonisin content (relevant only for maize flour and grit) was not taken into account in our calculations. While the change of fumonisin content as a result of processing operations was studied by several authors [21,22,23,24,25], and heating was reported to lead to some losses of the toxin, the results from different studies are variable [13]. Our approach might have led to a slight overestimation of exposure, taking into account that the effect of heating would lower the calculated intakes; however, this would not change our conclusions, considering that our results do not indicate serious health concern.

The fact that we took into account only those consumption days for which actual consumptions were registered also adds uncertainty. Given that maize-based commodities are non-staple commodities in Hungary, consumed only occasionally by the majority of the population, averaging the occasionally registered consumption values would be misleading. Similarly, including the zero-consumptions in our assessment would “dilute” the results.

However, it needs to be mentioned that current trends indicate an increase in gluten-free foods consumption, which is not strictly linked to the number of consumers intolerant to gluten. Regular consumers striving for healthy diets may as well choose maize-based foods. These facts highlight the importance of focusing more attention on these kinds of food products, considering that they also tend to be the focal commodities most highly contaminated with fumonisins.

As the consumption data were collected in 2009, certain changes might have occurred since then. In the case newer/more recent consumption data are published, repeating these evaluations would be of great value. In this regard, the consumption of different maize-based products could be studied in more detail. In our calculations, we linked the concentration data of an aggregated “maize-based, snack-like products” group to the consumption of an aggregated maize-based products group, including popped maize or extruded corn flakes. These calculations, however, could be refined by separately studying the consumptions of these specific products. Our measurement results indicate a relatively high contamination rate in this kind of commodity category.

## 3. Conclusions

Our calculations based on recent fumonisin analyses in maize-based foods and consumption data from a Hungarian survey produced comforting results. The calculated fumonisin intakes of the total population and of children consumers were well below the reference values established by JECFA and EFSA. The values were also in the same range or lower than the European exposure rates estimated by EFSA in 2014.

However, the recent trend of increasing the consumption of alternative, “healthy” foods, including maize-based commodities, needs to be monitored. Our results suggest that maize-based products may indeed be contaminated by fumonisins. Therefore, monitoring of fumonisins’ levels and the frequent re-evaluation of their dietary intakes with the best available data are recommended.

## 4. Materials and Methods

### 4.1. Sampling

Maize-based products were purchased from the Hungarian market in three metropolitan regions, i.e., Kaposvár (n = 276), Budapest (n = 29), and other cities, e.g., Debrecen, Keszthely, Székesfehérvár (n = 21). Commercial products were collected from supermarkets, retail shops, and pharmacies. A total amount of 326 samples purchased in 15 months (from August 2017 to November 2018) included maize flour (64), maize grits (62), corn flakes (64), canned maize (18), and other maize-based, snack-like products (85, extruded corn bread, tortilla chips, popcorn, nacho, maize chips, etc.). Beside these, 16 white and 10 brown rice and 7 rice-based products were also sampled. All information about the samples (i.e., producer, distributor, country of origin) was obtained from the products’ labels and recorded. Samples were randomly selected, collecting as many as possible leader and minor brands available on the market.

### 4.2. Laboratory Analysis

#### 4.2.1. Chemicals

Fumonisin B_1_ (FB_1_) and B_2_ (FB_2_) were purchased from Merck-Sigma Aldrich (St. Louis, LO, USA). HPLC–MS-grade acetonitrile and water were purchased from Carl Roth GmbH (Karlsruhe, Germany), HPLC–MS-grade acetic acid was purchased from Merck (Darmstadt, Germany).

#### 4.2.2. Sample Preparation

Dry solid samples were ground using an ETA^®^ Vital Blend II blender (ETA a.s., Praha, Czech Republic). Then, 5 g of sample was vortexed for 1 min with 20 mL of acetonitrile/water (50:50) on a VELP ZX-3 desktop vortex (Velp, Usmate, Italy) and 0.1% acetic acid and extracted for 60 min at 420 rotations/min speed on a horizontal desktop shaker (Edmund Bühler SM30A model, Bodelshausen, Germany). The supernatant of the extracted sample was centrifuged for 10 min at 14,000 rpm, and 4 °C. Aliquots of 10 µL internal standard solutions (^13^C-FB_1_, 6 µg/mL) were added to 970 µL aliquots of the supernatant of the centrifuged sample. The mixture was analyzed with LC–MS.

#### 4.2.3. High-Performance Liquid Chromatography

Liquid chromatography and mass spectrometry (LC–MS) analysis were performed with a Shimadzu Prominence UFLC separation system equipped with an LC–MS-2020 single quadrupole (ultra-fast) liquid chromatographer–mass spectrometer (Shimadzu, Kyoto, Japan) with electrospray source. Optimized mass spectra were obtained with an interface voltage of 4.5 kV and a detector voltage of 1.05 kV in negative mode and 1.25 kV in positive mode. Samples were analyzed on a Phenomenex Kinetex 2.6 μm XB-C18 100 Å column (100 mm × 2.1 mm, Phenomenex, Torrance, CA, USA). The column temperature was set to 40 °C; the flow rate was 0.3 mL/minute. Gradient elution was performed using LC–MS-grade water (Carl Roth GmbH, Karlsruhe, Germany) (eluent A) and acetonitrile (Carl Roth GmbH, Karlsruhe, Germany) (eluent B), both acidified with 0.1% acetic acid (Merck, Darmstadt, Germany). Then, 5 µL of each samples were analyzed with the gradient: (0 min) 5% B, (3 min) 60% B, (8 min) 95% B, followed by a holding time of 3 min at 95% eluent B and 2.5 min column re-equilibration with eluent 5% B. FB_1_ (diluted from 10 mg/L) standard solutions were used as references. MS parameters: source block temperature 90 °C; desolvation temperature 250 °C; heat block temperature 200 °C; drying gas flow 15.0 L/minute. Detection was performed using selected ion-monitoring (SIM) mode.

Detection (LOD) and quantification (LOQ) limits were 31 and 93 µg/kg for FB_1_ and 51 and 154 µg/kg for FB_2_.

For the calculation of LOD and LOQ, nine calibration points (0.1 µg/kg; 0.5 µg/kg; 1 µg/kg; 5 µg/kg; 10 µg/kg; 50 µg/kg; 100 µg/kg; 500 µg/kg; 1000 µg/kg) were measured, and the LOD and LOQ were calculated using the STHIBAYX function in Microsoft^®^ Excel (Version 2013, Microsoft Corporation, Redmond, WA, USA). The slope of the calibration curve was determined using the nine calibration points.

$$LOD=\left(\mathrm{Peak}\;\mathrm{area}\;1,\;\mathrm{Peak}\;\mathrm{area}\;2,\;\ldots .;\;\mathrm{Concentration}\;1,\;\mathrm{Concentration}\;2,\ldots .\right)\cdot \frac{3,3333}{\mathrm{Slope}\;\mathrm{of}\;\mathrm{calibration}\;\mathrm{curve}}$$

$$LOQ=\left(\mathrm{Peak}\;\mathrm{area}\;1,\;\mathrm{Peak}\;\mathrm{area}\;2,\;\ldots .;\;\mathrm{Concentration}\;1,\;\mathrm{Concentration}\;2,\ldots .\right)\cdot \frac{10}{\mathrm{Slope}\;\mathrm{of}\;\mathrm{calibration}\;\mathrm{curve}}$$

Ms Excel 2010 was used for the evaluation of the results.

### 4.3. Analysis of the Measurements and Correlation between FB_1_ and FB_2_ Concentrations

Main descriptive statistics (mean, maximum, 95th percentile) of the measured fumonisin contamination of the analyzed commodities were used. The measurement results were also characterized regarding the number of non-detected/not measurable values and the samples with fumonisin content exceeding the regulatory limit.

To take into account the uncertainty derived from the non-detected (<LOD) and detected but not measurable values (<LOQ), two scenarios were considered. First, to account for the worst-case option, assuming the highest possible concentration of these non-numerical values, LOD was inserted for values <LOD, and LOQ was inserted for values <LOQ, for both fumonisin B_1_ and B_2_ results. This was termed the upper-bound scenario. To illustrate with numbers, 0.031 mg/kg and 0.051 mg/kg were substituted for values of FB_1_ and FB_2_ <LOD, respectively, and 0.093 mg/kg and 0.154 mg/kg were substituted for values of FB_1_ and FB_2_ <LOQ, respectively.

To account for an optimistic scenario, assuming the lowest possible concentration, values <LOD were replaced with 0, and values <LOQ were replaced with the relevant LOD. This scenario was termed the lower-bound scenario. To illustrate with numbers, 0 was inserted for values of both fumonisins <LOD, and 0.031 mg/kg and 0.051 mg/kg were inserted for values of FB_1_ and FB_2_ <LOQ, respectively. Obviously, in the case of values >LOQ, the measured numerical values were used directly in all scenarios.

The possible correlation between fumonisin B_1_ and B_2_ contents in the samples was also analyzed, calculating the correlation coefficients. Besides considering only the corresponding numerical values of FB_1_ and FB_2_, we also analyzed a larger sample set, including those samples for which a numerical FB_1_ value was accompanied by a detected but not measurable (i.e., between LOD and LOQ) FB_2_ result. Lower- and upper-bound scenarios were calculated for these sample results as well.

### 4.4. Food Consumption Data

Consumption data were obtained from a survey carried out jointly by the Hungarian Food Safety Office (HFSO) and the Hungarian Central Statistical Office in 2009. Three-day dietary record data were collected from 4992 consumers, providing overall 14,976 daily food consumption data, including those of 934 children (aged below 18).

Relevant consumptions of maize products were recorded specifically for maize flour, maize grits, corn flakes, sweet maize, canned maize, frozen maize, extruded corn flakes, popped maize (with and without oil), and cheese-flavored popped maize. These products, and consequently their consumptions, were linked to the analyzed products, in order to perform intake calculations based on the concentration and consumption data of these specific commodities. Table 7 presents the commodities analyzed in relation to those consumed.

The maize-based, snack-like products measured mainly consisted of different types of snacks produced from maize, including nacho, tacoshells, corn flips, tortilla chips, extruded maize snack, etc. Although they had different compositions, they were dealt with in one aggregated commodity group called maize-based, snack-like products, as their compositions were not specified in the consumption data.

The effect of processing was not taken into account for two reasons. First, the effect of milling was not relevant, as the analytical measurements and consumptions were both recorded for milled maize products, enabling a direct linkage between them. On the other hand, the effect of heating was relevant for maize flour and maize grits; however, further studies would be necessary to conclude on the quantitative effect of heating, based on the literature.

We considered only the consumption days for which consumption of the selected foods was reported. Given that maize-based products are not consumed daily, including those individuals who did not report any consumption of these foods would unrealistically dilute our data. The main statistical parameters of the consumption data are summarized in Table 8. 

### 4.5. Risk Assessment Approach

Risk assessment was performed by semi-probabilistic means, by considering the consumption values as a distribution, since there were exact individual food consumption data available. The concentration values of FB_1_ and FB_2_ were summed in each sample and considered accordingly in further calculations.

For each food commodity category, the average concentration was calculated for both the LB and the UB scenarios. These values were then multiplied by the relevant individual consumption data one-by-one, resulting in the relevant calculated fumonisin intake values for each individual consumption of each commodity.

The daily individual intakes calculated from each commodity category were then summed for each individual, resulting in the summed individual daily fumonisin intake from all the selected foods. The resulting distribution of individual total daily fumonisin intakes could then be further studied on a population level. Average and high (95 percentile) values were calculated to determine the fumonisin intake of average and high consumers. These calculations were also applied for the children population of consumers. Finally, the resulting values were compared to the reference values established by JECFA [11] and EFSA [15].

To estimate the commodity contributions to the summed intake estimated from the analyzed commodities, the population average intake from each commodity was calculated separately and then compared to the average summed intake, resulting in the proportion of contribution of each commodity.

## Figures and Tables

**Table 1:** Maximum limits (µg/kg) for fumonisins established by Commission Regulation (EC) No 1881/2006.

2.6	Fumonisins	Sum of B_1_ and B_2_
2.6.1	Unprocessed maize, with the exception of unprocessed maize intended to be processed by wet-milling	4000
2.6.2	Maize intended for direct human consumption, maize-based foods for direct human consumption, with the exception of foodstuffs listed in 2.6.3 and 2.6.4	1000
2.6.3	Maize-based breakfast cereals and maize-based snacks	800
2.6.4	Processed maize-based foods and baby foods for infants and young children	200
2.6.5	Milling fractions of maize with particle size >500 µm, falling within CN code 1103 13 or 1103 20 40, and other maize milling products with particle size >500 µm not used for direct human consumption, falling within CN code 1904 10 10	1400
2.6.6	Milling fractions of maize with particle size ≤500 µm, falling within CN code 1102 20, and other maize milling products with particle size ≤500 µm not used for direct human consumption, falling within CN code 1904 10 10	2000

**Table 2:** Classification of the samples analyzed in this study in relation to fumonisins’ limit of detection (LOD), limit of quantification (LOQ) *, and the regulatory limit.

		Fumonisin B_1_			Fumonisin B_2_			
Commodity Category	Nr	<LOD	LOD-LOQ	>LOQ	<LOD	LOD-LOQ	>LOQ	Samples Over the Regulatory Limit (Regarding FB_1_ + FB_2_ Content)
Maize flour	64	12	19	33	48	10	6	1 (1.6%)
Maize grits	62	18	18	26	51	7	4	2 (3.2%)
Corn flakes	64	37	19	8	63	1	0	0
Canned maize	18	17	0	1	17	1	0	0
Maize-based, snack-like products	85	48	20	17	78	5	2	2 (2.4%)
Brown rice	10	9	1	0	10	0	0	0
White rice	16	14	2	0	16	0	0	0
Rice-based products	7	7	0	0	7	0	0	0

* LOD and LOQ for FB_1_: 0.031 and 0.093 mg/kg, LOD and LOQ for FB_2_: 0.051 and 0.154 mg/kg. Nr: number of samples.

**Table 3:** Descriptive statistics of the results obtained for in the different maize-based food categories.

		Fumonisin B_1_ (mg/kg)				Fumonisin B_2_ (mg/kg)			
Commodity Category	Nr	Mean (LB)*	Mean (UB)*	P95 (LB)	Max	Mean (LB)	Mean (UB)	P95 (LB)	Max
Maize flour	64	0.17	0.20	0.66	1.46	0.05	0.10	0.32	0.73
Maize grits	62	0.13	0.16	0.50	1.96	0.03	0.08	0.12	0.58
Corn flakes	64	0.03	0.07	0.12	0.46	na	na	Na	na
Canned maize	18	0.01	0.04	0.03	0.20	na	na	Na	na
Maize-based, snack-like products	85	0.07	0.10	0.36	1.10	0.01 **	0.06 **	0.05 **	0.17 **
All maize samples analyzed	293	0.09	0.12	0.46	1.96	0.02	0.07	0.05	0.73

* Method of mean calculation: Results below the LOD and between LOD and LOQ were taken into account in two ways. In the lower-bound (LB) scenario, 0 and LOD were inserted for values below LOD and between LOD and LOQ, respectively. In the upper-bound (UB) scenario, LOD and LOQ were inserted for values below LOD and between LOD and LOQ, respectively. ** There were only two measured values for the concerned food category and mycotoxin; na: not applicable. Nr: number of samples.

**Table 4:** Calculated mean and 95 percentile (P) for fumonisin intakes (µg/kg bw/day). The percentage of European Food Safety Authority (EFSA) tolerable daily intake (TDI) is included in brackets.

Intake Level	All Consumers (LB Scenario)	All Consumers (UB Scenario)	Children (LB Scenario)	Children (UB Scenario)
Mean intake	0.045 (4.5%)	0.120 (12.0%)	0.056 (5.6%)	0.167 (16.7%)
95P intake	0.182 (18.2%)	0.396 (39.6%)	0.244 (24.4%)	0.537 (53.7%)

**Table 5:** Summary of estimated intakes (µg/kg bw/day) in comparison with EFSA estimations and health-based guidance values.

Comparison with EFSA Results and Reference Intakes	Children’s Mean Exposure		Children’s High Exposure		Adults’ Mean Exposure *		Adults’ High Exposure *	
	LB Scenario	UB Scenario	LB Scenario	UB Scenario	LB Scenario	UB Scenario	LB Scenario	UB Scenario
Our results	0.056	0.167	0.244	0.537	0.045	0.120	0.182	0.396
EFSA 2014	0.17–1.52	0.47–2.11	0.54–3.44	1.09–4.39	0.03–0.81	0.15–1.19	0.08–1.76	0.31–2.30
% of JECFA PMTDI	2.8	8.4	12.2	26.9	2.2	6.0	9.1	19.8
% of EFSA TDI	5.6	16.7	24.4	53.7	4.5	12.0	18.2	39.6

* All (adult + children) consumers included in our calculations. JECFA: Joint FAO/WHO Expert Committee on Food Additives, PMTDI: provisional maximum tolerable daily intake.

**Table 6:** Contribution of different commodities to total fumonisin intake from maize-based products.

Scenario	Average Intake and % Contribution	Maize Flour	Maize Grits *	Corn Flakes	Canned Maize	Maize-Based, Snack-Like Products	All Maize-Based Products
Total population (LB scenario)	average intake of FB (µg/kg bw/day)	0.013	0.001	0.007	0.005	0.019	0.045
	% contribution	29.1%	2.1%	15.1%	10.4%	43.3%	100%
Total population (UB scenario)	average intake of FB (µg/kg bw/day)	0.018	0.001	0.027	0.033	0.041	0.120
	% contribution	14.8%	1.2%	22.3%	27.4%	34.3%	100%
Children (LB scenario)	average intake of FB (µg/kg bw/day)	0.005	0	0.011	0.006	0.034	0.056
	% contribution	8.2%	0.0%	20.2%	11.2%	60.4%	100%
Children (UB scenario)	average intake of FB (µg/kg bw/day)	0.006	0	0.045	0.044	0.072	0.167
	% contribution	3.7%	0.0%	26.8%	26.5%	43.0%	100%

* Data are shown, but conclusions cannot be made due to extremely low registered consumption.

**Table 7:** Linking of analyzed values to consumed maize commodity categories by commodity name.

Commodities with Analyzed Fumonisin Content	Commodities Present in the Food Consumption Database	Number of Consumption Days (Out of 14,976 Data)	Number of Consumption Days, Children (Out of 2802 Data)
Maize flour	maize flour	54	5
Maize grits	maize grits	4	0
Corn flakes	corn flakes	399	137
Canned maize	canned maize, sweet maize, frozen maize	176	45
Maize-based, snack-like products	maize popped, extruded corn flakes, cheese flavored popped maize	102	34

**Table 8:** Main statistical parameters of the consumption data.

Population	Parameter	g/Capita/Day Consumptions					g/kg bw/Day Consumptions				
		Maize Flour	Maize Grits	Cornflakes	Canned Maize	Maize-Based, Snack-Like Products	Maize Flour	Maize Grits	Cornflakes	Canned Maize	Maize-Based, Snack-Like Products
All consumers’ data	count	54	4	399	176	102	54	4	399	176	102
	mean	47.8	70.0	16.9	73.9	83.7	0.75	1.04	0.35	1.36	1.78
	P95	96.0	111.0	50.0	200.0	200.0	1.52	1.64	0.98	3.72	4.54
	maximum	220	120	100	330	400	3.67	1.76	4.55	11.54	11.11
Children’s data	count	5	0	137	45	34	5	0	137	45	34
	mean	38.4	na	20.4	61.0	74.6	0.89	na	0.57	2.12	2.69
	P95	53.2	na	60.0	150.0	150.0	1.33	na	1.96	5.52	6.75
	maximum	54	na	100	200	250	1.42	na	4.55	11.54	11.11

Na.: not applicable.
